# FLDO-LKNet: An Efficient Method for Segmenting Stone Cells in Rubber Tree Bark with Robustness to Staining Differences and Morphological and Scale Changes

**DOI:** 10.3390/plants15142178

**Published:** 2026-07-16

**Authors:** Yeling Peng, Yuanyuan Zhang, Hao Zeng, Yiqing Zeng, Meixi Pan, Zhongyang Peng, Yang Liu, Yongling Xia, Peng Wang, Mingfang He, Yaowen Hu

**Affiliations:** 1 College of Computer and Mathematics, Central South University of Forestry and Technology, Changsha 410004, China; 20232322@csuft.edu.cn (Y.P.); 20253975@csuft.edu.cn (H.Z.); t20162306@csuft.edu.cn (M.H.); 2 State Centre for Rubber Breeding, Rubber Research Institute, Chinese Academy of Tropical Agricultural Sciences, Haikou 571101, China; zhangyuanyuan@catas.cn; 3 College of Mechanical and Intelligent Manufacturing, Central South University of Forestry and Technology, Changsha 410004, China; 20232170@csuft.edu.cn; 4 College of Electronic Information and Physics, Central South University of Forestry and Technology, Changsha 410004, China; 20223881@csuft.edu.cn (M.P.); 20241318@csuft.edu.cn (Z.P.); 17570886178@163.com (Y.L.); 5 Department of Electronic Engineering, Tsinghua University, Beijing 100084, China; huyaowen_12345@163.com

**Keywords:** semantic segmentation, stone cell segmentation, rubber tree, deep learning, image segmentation

## Abstract

Stone cells are an important structural component of rubber tree bark and are closely associated with traits such as cracking propensity, bark hardness, stress tolerance and latex production. However, high-precision segmentation methods that are robust to staining variations, morphological diversity, and scale changes are still missing. To address these challenges in stone-cell histological images, such as the inconsistent staining intensity, the diverse shapes and sizes of cells, and the interference of cell debris, we proposed an automated semantic segmentation network called FLDO-LKNet, which formulates stone-cell delineation as a pixel-wise semantic segmentation task. Specifically, we introduce an LDFE module to recalibrate backbone features at the channel level, thereby mitigating the effects of staining differences. A KCFA attention mechanism is designed to better capture complex morphology and scale variation. In addition, we develop an FLDO optimization algorithm with a performance-feedback-based dynamic learning rate adjustment strategy to enhance robustness against training instability caused by debris interference. We further construct a dataset of 1084 stone-cell images collected from CATAS to support model training and evaluation. Experimental results demonstrate that FLDO-LKNet achieves 75.18% mIoU, 98.5% accuracy, and 82.21% sensitivity. Overall, as a dedicated semantic segmentation network, the proposed method enables high-precision pixel-level segmentation of stone cells, which may facilitate subsequent studies of stone-cell development and genetic functions and shows potential for agricultural applications.

## 1. Introduction

Stone cells are an important structural component of the bark of the rubber tree. Their spatial distribution and degree of accumulation are closely associated with key traits such as bark cracking, tissue hardness, stress resistance, and latex yield [1,2]. Therefore, they are regarded as an anatomical trait with potential value for breeding. However, current studies on stone cells still rely mainly on manual observation or coarse statistical analysis, which limits their application in large-scale phenotyping and constrains deeper investigation into their genetic basis in relation to latex yield, bark integrity, and stress tolerance. Accordingly, developing a highly accurate method for stone cell segmentation in rubber tree bark is of considerable theoretical and practical significance.

In recent years, the rapid development of deep learning has provided strong support for advances in plant science research [3,4,5,6,7,8]. In the early stage, cell-image segmentation relied mainly on traditional image-processing algorithms [9,10,11,12], which partitioned image regions according to differences in pixel intensity distributions or gradients. However, these methods performed poorly when cell boundaries were indistinct, image noise was substantial, or targets overlapped or occluded one another. Illumination variation, in particular, has long been recognised as a major difficulty for agricultural image segmentation: for instance, Wang et al. [13] combined wavelet-based illumination normalisation with a Retinex-enhanced K-means algorithm to achieve robust fruit segmentation under varying illumination. Nevertheless, such handcrafted pipelines still depend on empirical parameter tuning and generalise poorly to the coupled staining- and illumination-induced appearance variation observed in stone-cell histological images, which motivates the learnable feature-recalibration design adopted in this work.

With the advancement of deep learning, CNN-based methods achieved strong performance in image segmentation [14,15,16,17,18]. FCN was the first to replace the fully connected layers in conventional CNNs with convolutional layers and introduce upsampling operations, thereby enabling pixel-level classification for images of arbitrary size. U-Net proposed an encoder-decoder architecture and introduced skip connections between corresponding layers, effectively integrating features at different scales. U-Net++ further incorporated nested and dense short and long skip pathways, while also allowing redundant branches to be pruned. The DeepLab series introduced atrous convolution and the atrous spatial pyramid pooling (ASPP) module to enlarge the receptive field and capture multi-scale contextual information, thereby improving segmentation performance for objects of varying sizes.

After Transformers were introduced into computer vision [19], several Transformer-based methods also demonstrated strong performance [20,21,22,23,24]. However, these methods generally suffered from high computational complexity. More recently, SS2D-based methods and KAN-based methods, as physically inspired models, have attracted increasing attention because they can model complex spatial relationships and improve segmentation performance. These methods often surpass the performance of standard deep learning models in the case of data scarcity or high variability because of the incorporation of physical constraints. VM-UNet [25] integrated a state-space model (SSM) into the U-Net structure, significantly reducing parameter and computational costs while maintaining segmentation efficiency. UltraLight VM-UNet [26] achieved more network optimisation, thus a better trade-off between efficiency and accuracy in resource-constrained scenarios. U-KAN [27] integrated KAN with a U-shaped architecture and introduced multidimensional attention mechanisms, and multi-scale decoder outputs, which improved representational ability and segmentation performance with relatively low parameter budget.

**Figure 1:**
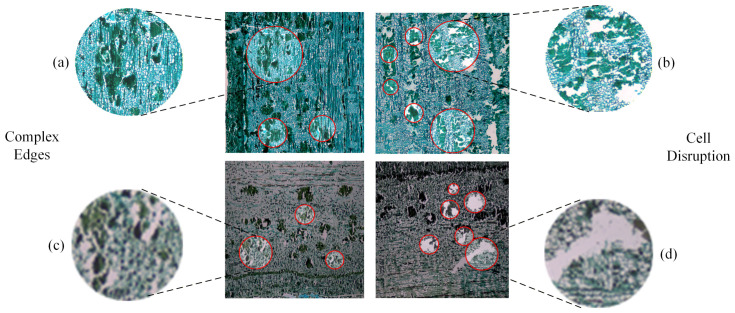
Selected dataset samples.

There are a number of unique difficulties with rubber tree stone-cell images. First, the cell walls are thick and irregular, which leads to uneven staining intensity, e.g., panels (a) and (b) in Figure 1 are more deeply stained than panels (c) and (d). Second, as shown in Figure 1a,c, the cell boundaries are morphologically complex and vary greatly in scale. Third, these images often include a lot of cellular debris, as shown in Figure 1b,d. These characteristics are not well addressed by the above methods, and their performance in the rubber tree stone-cell analysis is limited.

The current rubber tree stone-cell segmentation has four main obstacles: **1. Lack of datasets**: The public datasets of rubber tree stone cells are extremely limited. The existing datasets are insufficient in terms of sample size and sample diversity and the model training lacks sufficient data support. **2. Staining variation interference**: Neural networks frequently confuse staining variation (a broad term for the variation in colour depth, brightness and contrast that exists across images due to the combined effect of staining conditions and illumination intensity) with discriminative cues, leading to a deterioration of the ability to model fine-grained features such as the stone-cell texture accurately. **3. High variability in cell shape and size**: The cell boundaries are more difficult to distinguish and thus more difficult segmentation due to the high variability in both the shape and size of stone cells in rubber tree bark and easy introduction of artefacts like cell debris during sectioning. **4. Cell debris increases sensitivity to the learning rate**: Cell debris introduces false edge responses and interference from non-target details, making it difficult for the model to learn stable representations of target boundaries and thereby increasing noise in gradient estimation and destabilizing parameter updates during training. Consequently, the reliability of the gradient signal is reduced, the range of learning rates that supports stable training becomes narrower (an excessively large learning rate may cause oscillation or even divergence, whereas an excessively small one leads to slow convergence), and the model becomes more sensitive to the initial learning-rate setting.

To address the above issues, **1. We constructed a new rubber tree stone-cell dataset**: Owing to the lack of stone-cell data, and under the guidance of experts from the Rubber Research Institute of the Chinese Academy of Tropical Agricultural Sciences (CATAS), a single trained annotator carried out the precise annotation of rubber tree stone cells, thereby constructing a high-quality semantic segmentation dataset. **2. We propose a Lighting Calibration and Detail Feature Enhancement (LDFE) module to mitigate interference from staining variation**: This module performs channel-wise adaptive linear recalibration of backbone features to compensate for illumination and staining bias, reduce the interference caused by staining variation, and enhance the network’s ability to extract fine-grained features such as stone-cell texture. **3. We propose Kalman Cross-layer Fusion Attention (KCFA) to address variation in cell morphology and size**: KCFA first extracts multi-scale features to adapt to stone cells of different sizes, then strengthens boundary responses through joint channel–spatial modeling, and finally performs a Kalman-inspired fusion that treats the high-level semantic features from the decoder as an analogue of the prior and the detailed features from the encoder as an analogue of the observation, thereby accurately supplementing boundary details in morphologically complex regions under semantic guidance. **4. We propose a Feedback-guided Learning-rate Desensitization Optimizer (FLDO) to address the increase in learning-rate sensitivity caused by cell debris**: Built upon adaptive gradient estimation, FLDO introduces a performance-feedback-driven dynamic learning-rate regulation mechanism to suppress interference from cell debris and reduce sensitivity to the initial learning-rate setting, thereby effectively improving training stability.

The main contributions of this study are as follows:

1. We constructed a high-quality dataset that, to the best of our knowledge, is the first dataset specifically developed for rubber tree stone cells.
2. We propose a new model, FLDO-LKNet, which consists of the following components:
  (a) LDFE, which performs channel-wise recalibration of backbone features to reduce interference from staining variation;
  (b) KCFA, which addresses the complex morphological and scale variation of stone cells through a Kalman-style cross-layer fusion mechanism;
  (c) FLDO, which introduces a performance-feedback-driven dynamic learning-rate regulation mechanism to address the increased learning-rate sensitivity caused by cell debris.
3. The proposed FLDO-LKNet achieved an mIoU of 75.18% and a DSC of 82.36%, outperforming existing methods in overall segmentation performance.

## 2. Materials and Methods

### 2.1. Materials

Bark samples for stone-cell segmentation were obtained from three rubber tree populations in Hainan Province, China [3]. The materials included two clonal populations and one hybrid progeny population. The clonal materials were derived from 8 clones planted in 2013 and sampled in 2021 and from 11 clones planted in 2006 and sampled in 2022. Both clonal populations were cultivated at 3.0 m × 7.0 m spacing, and 5 and 3 trees per clone were randomly sampled, respectively. In addition, 253 hybrid progenies planted in 2016 were sampled during 2020–2021 from a plantation established at 1.5 m × 3.0 m density.

Bark blocks (0.5 cm^2^) were fixed in 70% ethanol and prepared for anatomical examination through dehydration, paraffin embedding, sectioning, and staining [28,29]. After sectioning to 15 μm, the slides were deparaffinized, stained with Fast Green, and mounted for microscopic examination. Image acquisition was carried out with three microscope–camera combinations corresponding to the three groups: Olympus BX53 and Olympus CX43 microscopes (Olympus Corporation, Tokyo, Japan) paired with VP700c and LV2000 cameras, respectively, and a Leica DMBL microscope with a Leica DFC550 camera (Leica Microsystems, Wetzlar, Germany). No fewer than three images were collected for each sample.

### 2.2. Dataset

**Figure 2:**
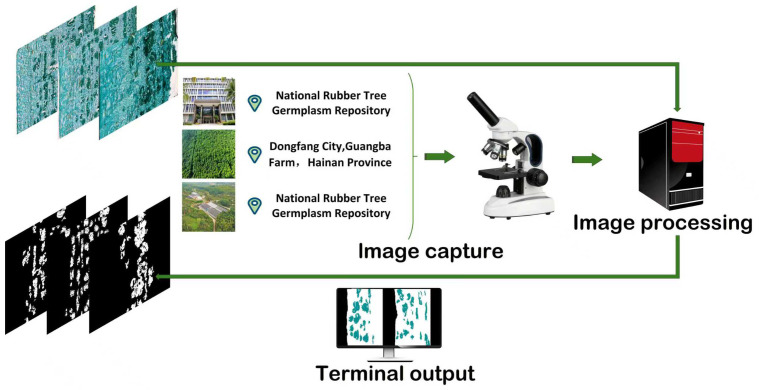
Schematic diagram of the rubber tree sclereid segmentation system.

Establishing a suitable image dataset is crucial for the quantitative analysis of rubber tree phenotypes, as well as for improving, validating, and benchmarking rubber tree stone cell segmentation algorithms [30,31]. In total, 3200 paraffin-embedded cross-sectional bark images were collected, from which 1084 representative images were ultimately selected to construct the rubber tree stone cell dataset (Figure 2). The reduction from the 3200 collected images to the final 1084 images followed an explicit two-step sampling rule. First, a quality-control screening was applied to discard images that were out-of-focus or blurred, unevenly or abnormally stained, affected by sectioning artefacts (e.g., tissue folding, tearing, or air bubbles), or nearly duplicated with adjacent serial sections of the same sample. Second, from the quality-qualified images, a stratified selection was performed to maintain balanced coverage across the three populations (the two clonal populations and the hybrid progeny population), their corresponding sampling years and developmental stages, and the three microscope–camera acquisition combinations described in the Section 2.1, so that no single cultivar or acquisition condition dominated the dataset. The dataset was designed to capture the large variation in stone cell morphology and abundance between cultivars and developmental stages. Specifically, the dataset comprised images from two clonal populations and one hybrid progeny population: an eight-clone population planted in 2013 and sampled in 2021; an eleven-clone population planted in 2006 and sampled in 2022; and 253 hybrid progenies planted in 2016 and sampled in 2020–2021. The 1084 annotated images were split into 884 images for model development and 200 images for independent testing. Within the 884 development images, 15% (133 images) were randomly separated as the validation set, while the remaining 751 images (approximately 85%) were used for training. The validation subset was used only for performance feedback, learning-rate regulation and model selection during training. The independent test set was not used in training or validation but only for the final evaluation of the segmentation performance.

**Figure 3:**
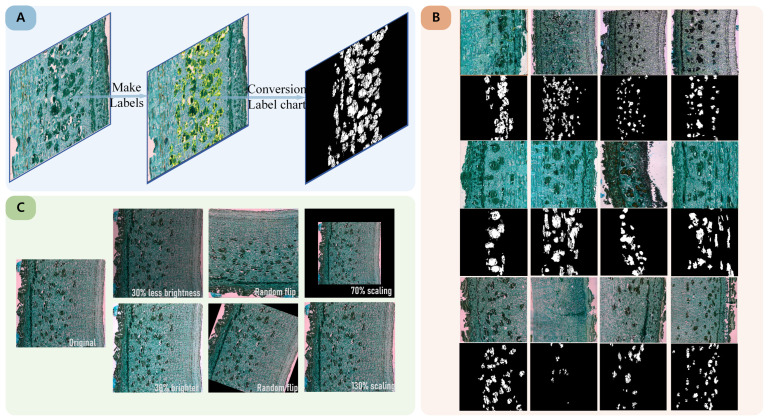
Illustration of the annotation procedure and the enhancement applied to labels and original images: (**A**) annotation pipeline; (**B**) examples of raw and labeled images; (**C**) representative augmented samples.

Image annotation was carried out in Fiji (ImageJ). To ensure uniform labeling criteria across the entire dataset, all masks were produced by a single trained annotator following a standardized protocol under the guidance of the Rubber Research Institute team, and all resulting masks were subsequently reviewed and verified by the domain experts. All sections of bark were segmented for stone cells using the magic wand tool with a fixed threshold of 10. The same tolerance was applied uniformly across all three microscope–camera combinations; because the magic wand selects pixels by local similarity relative to the seed pixel within each image, this imposes a consistent relative selection criterion across differently-acquired images, and any residual setup-specific selection error is corrected by the subsequent manual marking and expert verification. This setting eliminated false selection in non-relevant areas and guaranteed stable delineation of target pixels. The regions of the sclereids that appeared as blanks due to imperfect sectioning were also identified and manually marked during annotation. These regions are labelled as foreground, as they correspond to genuine stone-cell locations; they constitute only a small minority of the foreground pixels and were not recorded as a separate category during annotation. Each annotated image was stored in a binary PNG file. After quality control and statistical filtering, 1084 high quality binary masks were obtained. The annotation pipeline is shown in Figure 3A and examples of raw and labelled images used for subsequent segmentation tasks are shown in Figure 3B.

Deep learning models typically perform better when trained on datasets with broad appearance diversity. To enhance variability and strengthen model generalization, several augmentation strategies were applied: (a) photometric adjustments, such as brightness changes to simulate varying illumination; (b) geometric operations, including horizontal mirroring; and (c) scale-based transformations, such as image resizing. Representative augmented samples are shown in Figure 3C. All final annotations were stored in PNG format. It should be noted that the photometric augmentation used here was deliberately limited to brightness variation. Accordingly, the robustness of the proposed method to staining-intensity and contrast differences is not attributed to photometric augmentation; rather, it is achieved architecturally through the LDFE module, which performs channel-wise recalibration of brightness and staining statistics, and it is evaluated on the real staining and illumination variation inherently present in our dataset, which was acquired from three different microscope–camera combinations across multiple populations and developmental stages. Broadening the photometric augmentation to contrast, saturation, and colour-jitter transformations is expected to further reinforce this robustness and is left for future work.

### 2.3. Method

#### 2.3.1. Overall Architecture

**Figure 4:**
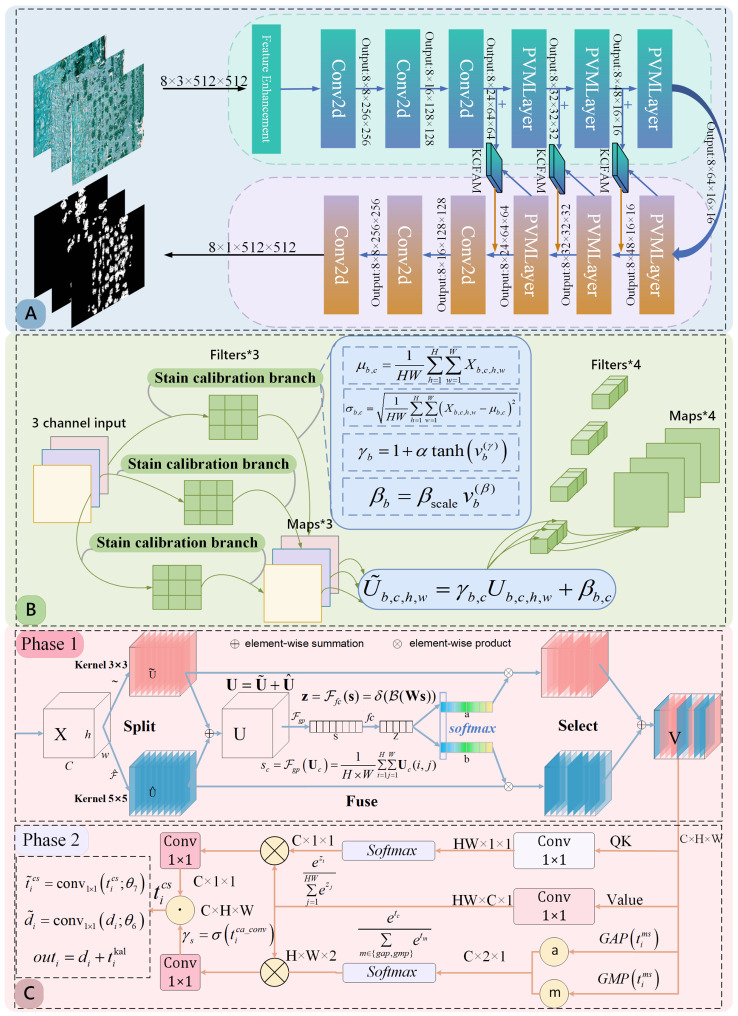
Overall structure of FLDO-LKNet: (**A**) overall architecture; (**B**) LDFE module; (**C**) KCFA module.

The overall architecture of the proposed network is shown in Figure 4A. First, we place LDFE before the encoder. LDFE applies channel-wise adaptive linear recalibration to the input features, which helps reduce the impact of illumination and staining variations on feature distributions. In addition, it supports local feature learning in the spatial domain and improves the representation of fine details. Next, KCFA is integrated into the cross-scale fusion unit at each decoder stage. In KCFA, high-level decoder features are treated as an analogue of the prior estimate, while the corresponding encoder features play the role of the observation. After feature dimensions are aligned, a Kalman-inspired residual update is performed, which strengthens the robustness of the model to changes in stone cell shape and size. During training, we propose the Feedback-guided Learning-rate Desensitization Optimizer (FLDO) to reduce the learning rate sensitivity caused by cell fragments. FLDO adjusts the learning rate scaling periodically based on performance feedback, which reduces dependence on the initial learning rate, stabilizes training, and supports faster convergence.

#### 2.3.2. Lighting Calibration and Detail Feature Enhancement Module (LDFE)

Early semantic segmentation models, such as FCN [30] and SegNet [31], mainly relied on encoders to extract hierarchical representations through stacked convolutional layers. Since no dedicated feature enhancement unit was explicitly introduced before or within the encoder, their adaptability to imaging variation was limited. To improve feature discriminability, later studies incorporated enhancement modules into different stages of the backbone or encoder, such as the FEM in FECANet [32], the AFE in FANet [33], and the CACEM in CAC-Net [34], all of which demonstrated the benefit of feature refinement for segmentation tasks.

As discussed in Section 1, rubber tree stone-cell images often exhibit noticeable appearance inconsistency across acquisition batches because illumination intensity and staining conditions are difficult to keep fully uniform. Existing enhancement modules, however, do not explicitly address this characteristic of histological bark images. To alleviate the interference caused by staining variation while preserving fine structural cues, we introduced a Lighting Calibration and Detail Feature Enhancement module (LDFE) before the encoder. Given an input feature map $X\in R^{B\times C\times H\times W}$, LDFE first applies a channel-wise 3×3 depth convolution to extract local spatial responses:

(1) $$U={\mathrm{DWConv}}_{3\times 3}\left(X;W_{\mathrm{dw}}\right),$$

where $U\in R^{B\times C\times H\times W}$ denotes the locally encoded feature map. Based on *U*, the mean and standard deviation of each channel are computed to characterize image-wise brightness and staining statistics:

(2) $$\mu_{b,c}=\frac{1}{HW}\sum_{h=1}^{H}\sum_{w=1}^{W}U_{b,c,h,w},$$

(3) $$\sigma_{b,c}=\sqrt{\frac{1}{HW}\sum_{h=1}^{H}\sum_{w=1}^{W}{\left(U_{b,c,h,w}-\mu_{b,c}\right)}^{2}}$$

The channel statistics of the three image channels are then concatenated into a staining description vector:

(4) $$s_{b}=\left[\mu_{b,1},\mu_{b,2},\mu_{b,3},\sigma_{b,1},\sigma_{b,2},\sigma_{b,3}\right]\in R^{6}.$$

To generate adaptive recalibration parameters, $s_{b}$ is fed into a single-hidden-layer multilayer perceptron:

(5) $$h_{b}=\mathrm{ReLU}\left(W_{1}s_{b}+b_{1}\right),$$

(6) $$v_{b}=W_{2}h_{b}+b_{2}\in R^{2C}.$$

The output vector is split into two *C*-dimensional components,

(7) $$v_{b}=\left[v_{b}^{\left(\gamma \right)},\;v_{b}^{\left(\beta \right)}\right],$$

from which channel-wise scaling and offset terms are obtained as

(8) $$\gamma_{b}=1+\alpha \tanh\;\left(v_{b}^{\left(\gamma \right)}\right),\;\beta_{b}=\beta_{\mathrm{scale}}\;v_{b}^{\left(\beta \right)}.$$

Here, α and $\beta_{\mathrm{scale}}$ are constants controlling recalibration magnitude. The original feature responses are then linearly corrected in a channel-adaptive manner:

(9) $${\tilde{U}}_{b,c,h,w}=\gamma_{b,c}\;U_{b,c,h,w}+\beta_{b,c}.$$

After illumination and staining recalibration, a 1×1 point-wise convolution is employed to strengthen inter-channel interaction and enhance the representation of texture and edge details:

(10) $$V={\mathrm{PWConv}}_{1\times 1}\left(\tilde{U};W_{\mathrm{pw}}\right).$$

Finally, batch normalization and nonlinear activation are applied to produce the output feature:

(11) $$\hat{V}=\mathrm{BN}\left(V\right),$$

(12) $$Y=\mathrm{ReLU}(\hat{V}).$$

In this way, LDFE suppresses staining-related feature perturbation through adaptive channel recalibration, while simultaneously improving the encoding of local textures and boundaries that are important for stone-cell segmentation. Its overall architecture is shown in Figure 4B, and the corresponding ablation results are presented in the Section 3.2.1.

#### 2.3.3. Kalman Cross-Layer Fusion Attention (KCFA)

To address the pronounced variation in shape and scale of rubber tree stone cells, we designed a Kalman Cross-layer Fusion Attention module (KCFA). Instead of directly combining encoder and decoder features by simple concatenation or summation, KCFA first constructs multi-scale contextual representations to cover stone cells of different sizes, and then introduces channel–spatial joint weighting to strengthen responses to complex boundaries while suppressing irrelevant interference. On this basis, by analogy with a Kalman measurement update, the high-level decoder feature is treated as an analogue of the semantic prior, whereas the corresponding encoder feature plays the role of a detailed observation. A Kalman-inspired prior–observation update is then performed to achieve adaptive cross-layer fusion, so that boundary details can be incorporated under semantic guidance in a more stable manner.

Specifically, let the encoder feature at the current scale be denoted by $t_{i}$ and the decoder feature by $d_{i}$. To enrich contextual information, $t_{i}$ is first processed by two parallel convolution branches with kernel sizes of 3×3 and 5×5, respectively. The outputs are denoted as $U_{1}$ and $U_{2}$, and their initial combination is written as

(13) $$U=U_{1}+U_{2}.$$

Global average pooling is then applied to obtain channel-wise statistical descriptors:

(14) $$s_{c}=\frac{1}{H\times W}\sum_{p=1}^{H}\sum_{q=1}^{W}U_{c}\left(p,q\right),$$

where $U_{c}\left(p,q\right)$ denotes the feature value at spatial position (p,q) in channel *c*. The descriptor is subsequently mapped by a fully connected transformation to generate adaptive branch weights:

(15) z=δ(B(Ws)),

followed by Softmax normalization:

(16) $$\alpha_{k}=\frac{e^{z_{k}}}{\sum_{j=1}^{K}e^{z_{j}}}.$$

where $\alpha_{1}$ and $\alpha_{2}$ are the normalized branch weights generated by the Softmax operation.

(17) $$F=\alpha_{1}\cdot U_{1}+\alpha_{2}\cdot U_{2}$$

where ⊙ denotes element-wise multiplication.

Although multi-scale context improves sensitivity to targets of different sizes, it remains insufficient for accurately delineating stone-cell boundaries in the presence of tissue debris and fragmented structures. Therefore, channel–spatial joint attention is further introduced to enhance the reliability of the observation feature. In the channel branch, global average pooling followed by a fully connected mapping is used to produce channel attention weights:

(18) $$a_{i}=\sigma \left(fc(GAP\left(t_{i}\right))\right),$$

where σ(·) denotes the sigmoid activation. In the spatial branch, a 3×3 convolution is applied to generate spatial attention weights:

(19) $$s_{i}=\sigma \left(conv_{3\times 3}\left(t_{i}\right)\right).$$

The two types of weights are sequentially imposed on $t_{i}$ to obtain the channel–spatial enhanced observation feature:

(20) $$t_{i}^{cs}=s_{i}⊙a_{i}⊙t_{i}.$$

For cross-layer fusion, by analogy with a Kalman measurement update, the decoder feature $d_{i}$ plays the role of the prior estimate and the enhanced encoder feature $t_{i}^{cs}$ plays the role of the observation. To ensure that both can be compared and updated within a shared feature space, 1×1 convolutions are first used for channel alignment:

(21) $${\tilde{t}}_{i}^{cs}=conv_{1\times 1}\left(t_{i}^{cs};\theta_{7}\right),\;{\tilde{d}}_{i}=conv_{1\times 1}\left(d_{i};\theta_{6}\right).$$

The aligned prior and observation are concatenated and passed through a 3×3 convolution followed by sigmoid activation to produce an adaptive Kalman-like gain:

(22) $$k_{i}=\sigma \left(conv_{3\times 3}\left(\left[{\tilde{d}}_{i},{\tilde{t}}_{i}^{cs}\right];\theta_{8}\right)\right).$$

Using this gain, the residual between observation and prior is selectively injected into the prior feature:

(23) $$t_{i}^{kal}={\tilde{d}}_{i}+k_{i}⊙\left({\tilde{t}}_{i}^{cs}-{\tilde{d}}_{i}\right).$$

Finally, the updated detailed representation is combined with the original decoder feature to generate the cross-layer fusion output:

(24) $$o_{i}^{out}=d_{i}+t_{i}^{kal}.$$

It should be noted that Equation (23) is adopted as a Kalman-inspired analogy rather than a literal Kalman filter. It shares the functional form of the Kalman measurement update, in which the posterior estimate equals the prior plus a gain applied to the innovation (here, the prior–observation residual ${\tilde{t}}_{i}^{cs}-{\tilde{d}}_{i}$); however, the gain $k_{i}$ is learned end-to-end by a convolutional layer conditioned on the aligned prior and observation, rather than being derived analytically from process- and measurement-noise covariances or from an explicit state-space model. In other words, $k_{i}$ serves as a spatially adaptive, data-driven counterpart of the Kalman gain, which avoids having to specify or estimate the noise statistics that are difficult to define for deep feature maps. Algebraically, Equation (23) is a spatially-varying gated residual fusion of the same form as attention-based feature fusion such as Attention U-Net [35], and it does not possess the defining properties of a genuine Kalman filter—an explicit noise model, a covariance-propagation equation, or optimality under a Gaussian assumption. We therefore do not claim a novel or principled Kalman formulation; the Kalman terminology is retained only to convey this prior–observation–update intuition. In this way, KCFA adaptively regulates the strength of detail injection according to the consistency between the semantic prior and the observed detail response. When the observation deviates from the prior because of fragmentation or debris interference, its contribution is suppressed to preserve contour coherence; when the two are consistent, informative boundary details are further strengthened. As a result, KCFA improves the robustness of cross-layer fusion to scale variation and complex morphology in rubber tree stone-cell segmentation. The overall structure of KCFA is shown in Figure 4C, and the corresponding ablation and comparison results are presented in the Section 3.2.2.

#### 2.3.4. Feedback-Guided Learning-Rate Desensitization Optimizer (FLDO)

Traditional optimisers like AdaGrad [36] and related gradient-based methods [37] are sensitive to learning-rate settings in rubber tree stone-cell segmentation. The training gradients are usually noisy under the disturbance of cell fragments, while the effective supervisory signal around stone-cell boundaries is relatively weak. A too large learning rate could result in oscillatory updates and a too small learning rate could slow down convergence. Adaptive optimisers such as AdamW [38] and AdaBelief [39] can automatically adjust step sizes, but they may still suffer from unstable updates or slow convergence in noisy gradient regions due to fragment interference. To address this issue, we propose a Feedback-guided Learning-rate Desensitization Optimizer (FLDO), which combines reliable gradient estimation with performance-feedback-based learning-rate regulation, thereby stabilizing training and improving convergence efficiency in the presence of fragment-induced noise.

Given the gradient $g_{t}$ at iteration *t*, FLDO first constructs a first-order moment $m_{t}$ and a second-order confidence moment $v_{t}$ to obtain a more reliable estimate of the optimization direction:

(25) $$m_{t}=\beta_{1}m_{t-1}+\left(1-\beta_{1}\right)g_{t},$$

(26) $$v_{t}=\beta_{2}v_{t-1}+\left(1-\beta_{2}\right){\left(g_{t}-m_{t}\right)}^{2}+\varepsilon ,$$

where $\beta_{1}$ and $\beta_{2}$ are decay coefficients and ε is a small constant for numerical stability. Bias-corrected estimates are then obtained as

(27) $${\hat{m}}_{t}=\frac{m_{t}}{1-\beta_{1}^{t}},\;{\hat{v}}_{t}=\frac{v_{t}}{1-\beta_{2}^{t}},$$

and the normalized reliable gradient is defined as

(28) $${\tilde{g}}_{t}=\frac{{\hat{m}}_{t}}{\sqrt{{\hat{v}}_{t}}}.$$

To reduce the influence of noisy gradients near blurred or fragmented stone-cell boundaries, FLDO further introduces a nonlinear gradient recalibration step. Specifically, the normalized gradient is first standardized as

(29) $$u_{t}=\frac{{\tilde{g}}_{t}-\mathrm{mean}\left({\tilde{g}}_{t}\right)}{\mathrm{std}({\tilde{g}}_{t})+\varepsilon }.$$

Assuming that *B* local response branches are used, the response of the *b*th branch is written as

(30) $$r_{t}^{\left(b\right)}=f_{b}\left(u_{t};\theta_{b}\right),$$

where $\theta_{b}$ denotes the parameters of branch *b*. Soft gating is then adopted to assign adaptive weights to different branches:

(31) $$\pi_{t}^{\left(b\right)}=\frac{\exp\;\left(h_{b}\left(u_{t};\psi_{b}\right)\right)}{\sum_{j=1}^{B}\exp\;\left(h_{j}\left(u_{t};\psi_{j}\right)\right)},$$

and the recalibrated gradient is obtained by weighted aggregation:

(32) $$\phi \left({\tilde{g}}_{t}\right)=\sum_{b=1}^{B}\pi_{t}^{\left(b\right)}r_{t}^{\left(b\right)}.$$

Intuitively, $u_{t}$ is the standardized reliable gradient (zero mean, unit variance), which makes the recalibration scale-invariant; each of the *B* branches $r_{t}^{\left(b\right)}$ is a lightweight nonlinear transform that reshapes the gradient in a different way—some emphasizing the consistent gradients along genuine stone-cell boundaries, others suppressing the sharp, isolated responses produced by cell fragments—and the soft-gating weights $\pi_{t}^{\left(b\right)}$ adaptively decide, at each step, how much to trust each branch, so that the aggregated $\phi ({\tilde{g}}_{t})$ acts as a learned, input-dependent denoising of the gradient. This design suppresses unreliable noisy responses while preserving informative gradient components related to stone-cell boundaries.

On this basis, FLDO introduces feedback-guided learning-rate regulation. To characterize the current optimization state and the learning difficulty in boundary regions, we define the state vector as

(33) $$s_{t}=\left[\mathrm{mean}\left(m_{t}\right),\mathrm{mean}\left(v_{t}\right),L_{t},\Delta L_{t},\mu_{t},\sigma_{t}^{2}\right],$$

where $L_{t}$ denotes the current loss, $\Delta L_{t}=L_{t}-L_{t-1}$ is the loss change between two consecutive iterations, and $\mu_{t}$ and $\sigma_{t}^{2}$ denote the mean and variance of gradients in the blurred-boundary pixel set, respectively. Let the action space be a discrete set of learning-rate scaling factors,

(34) $$A=\{a_{1},\ldots ,a_{K}\},$$

and each action corresponds to a positive scaling coefficient ρ(a). Then the learning rate at iteration *t* is dynamically determined as

(35) $$\alpha_{t}=\rho \left(a_{t}\right)\alpha_{0},$$

where $\alpha_{0}$ is the base learning rate.

To encourage learning-rate choices that improve segmentation quality, the reward is defined according to the validation performance change:

(36) $$r_{t}=\left(I_{t}-I_{t-T_{\mathrm{val}}}\right)+\left(D_{t}-D_{t-T_{\mathrm{val}}}\right),$$

where $I_{t}$ and $D_{t}$ denote the validation mIoU and Dice score at the current validation step, respectively, and $I_{t-T_{\mathrm{val}}}$ and $D_{t-T_{\mathrm{val}}}$ denote the corresponding values at the previous validation step. The action-value function is updated using the Bellman equation:

(37) $$Q\left(s_{t},a_{t}\right)\leftarrow \left(1-\eta_{Q}\right)Q\left(s_{t},a_{t}\right)+\eta_{Q}\left[r_{t}+\gamma_{Q}\max_{a^{\prime }}Q\left(s_{t+1},a^{\prime }\right)\right],$$

where $\eta_{Q}$ is the Q-learning update rate and $\gamma_{Q}$ is the discount factor, and the action is selected with an ϵ-greedy strategy:

(38) $$a_{t}=\left\{\begin{array}{cc} \arg\max_{a}Q\left(s_{t},a\right), & u>\epsilon ,\\ \mathrm{Uniform}(A), & u\leq \epsilon , \end{array}\right.$$

where u∼U(0,1).

Finally, parameter updating is performed by combining the feedback-controlled learning rate, the recalibrated gradient, and decoupled weight decay:

(39) $$g_{t}^{\prime }=\mathrm{clip}\;\left(\phi ({\tilde{g}}_{t}),-c,c\right),$$

(40) $$w_{t+1}=w_{t}-\alpha_{t}\left(g_{t}^{\prime }+\lambda w_{t}\right),$$

where *c* is the clipping threshold and λ is the decoupled weight decay coefficient.

Through this design, FLDO improves the robustness of optimization to learning-rate settings by coupling reliable gradient estimation with validation-performance feedback. When fragment interference causes boundary gradients to become noisy or unstable, the optimizer can suppress unreliable updates and adaptively regulate the learning rate, thereby maintaining more stable convergence and producing more reliable segmentation results for quantitative stone-cell analysis. The overall procedure of FLDO is summarized in Table 1, and the corresponding comparison and ablation results are presented in the Section 3.2.3. We note that the FLDO reward and the model-selection criterion both rely on the same validation feedback stream (validation mIoU and Dice), so the optimizer control and model selection are not fully independent. To prevent this coupling from biasing the reported results, all final performance metrics in this paper are computed on the independent test set, which is never used for reward computation, learning-rate regulation, or model selection; the validation stream therefore influences only how the model is trained and chosen, not how it is finally evaluated. A cleaner design that further disentangles these two roles—for example, by allocating two disjoint validation splits for optimizer feedback and for model selection, respectively—is a worthwhile refinement that we leave for future work.

The reinforcement-learning component of FLDO is configured as follows and kept fixed across all experiments: the Q-learning update rate is $\eta_{Q}=0.1$; the discount factor is $\gamma_{Q}=0.9$; the validation interval is $T_{\mathrm{val}}=94$ iterations (one epoch, given 751 training images and a batch size of 8), yielding approximately 300 policy updates over 300 epochs; the action space consists of |A|=5 discrete learning-rate scaling factors A={0.5,0.8,1.0,1.25,1.5}; and the action is selected by an ϵ-greedy policy whose exploration rate is annealed exponentially from $\varepsilon_{\max}=0.5$ to $\varepsilon_{\min}=0.05$ with a decay constant τ=100 update steps. These settings are summarized in Table 2.

### 2.4. Setup and Evaluation Metrics

All experiments in this study were conducted on a unified hardware and software platform to avoid potential bias in the performance evaluation of FLDO-LKNet caused by differences in experimental conditions. The dataset was divided into 884 development images and 200 test images, with each image resized to a resolution of 512 × 512 pixels. For feedback-guided learning-rate regulation, a validation subset (133 images, i.e., 15% of the 884 development images, leaving 751 images for training) was further separated from these development images and used only for performance feedback and model selection. The batch size was set to 8, and the model was trained for 300 epochs. A consistent and standardized software environment was used to ensure the reproducibility and comparability of the experimental results. The detailed hardware specifications and software configuration are listed in Table 3.

In practice, researchers often compare predicted segmentation results with ground-truth annotations by visual inspection. However, when the differences between them are not sufficiently obvious, subjective judgment alone becomes unreliable. Therefore, objective quantitative metrics are needed for evaluation. In this study, the segmentation results were assessed using mean Intersection over Union (mIoU), Sensitivity (Recall), Specificity, Accuracy, BceDSCLoss, and the Dice similarity coefficient (DSC). It should be noted that stone-cell segmentation is a highly class-imbalanced task: across the dataset, foreground (stone-cell) pixels account on average for only about 5.3% of each image (median 4.5%, ranging from 0.03% to 43.7%), corresponding to an approximate background-to-foreground ratio of 18:1. Under such imbalance, Accuracy and Specificity are dominated by the majority background class and tend to saturate at high values for all methods, so they offer limited discriminative power. For this reason, we regard the foreground-sensitive metrics—mIoU, DSC, and Sensitivity—as the primary basis for comparison, and report Accuracy and Specificity only for completeness.

mIoU measures the degree of overlap between the predicted target region and the ground-truth region, and is defined as

(41) $$\mathrm{mIoU}=\frac{1}{N}\sum_{i=1}^{N}\frac{TP_{i}}{TP_{i}+FP_{i}+FN_{i}},$$

where *N* denotes the number of classes, and $TP_{i}$, $FP_{i}$, and $FN_{i}$ represent the true positives, false positives, and false negatives for class *i*, respectively.

Sensitivity (Recall) represents the proportion of actual target pixels that are correctly identified by the model, and is calculated as

(42) $$\mathrm{Sensitivity}=\frac{TP}{TP+FN}.$$

Specificity reflects the proportion of correctly identified negative samples among all actual negative samples, and is defined as

(43) $$\mathrm{Specificity}=\frac{TN}{TN+FP}.$$

Accuracy indicates the proportion of correctly classified samples among all predictions, and is given by

(44) $$\mathrm{Accuracy}=\frac{TP+TN}{TP+FP+TN+FN}.$$

BceDSCLoss combines binary cross-entropy loss (BCE loss) and Dice loss (DSCLoss), with two weighting coefficients, $w_{b}$ and $w_{d}$, used to balance the contribution of each term. It is formulated as

(45) $$\begin{array}{cc} \mathrm{BceDSCLoss} & =w_{b}\left[-\frac{1}{N}\sum_{i=1}^{N}\left(t_{i}\log p_{i}+\left(1-t_{i}\right)\log\left(1-p_{i}\right)\right)\right]\\ \; & \;+w_{d}\left(1-\frac{2\sum_{i=1}^{N}p_{i}t_{i}+\mathrm{smooth}}{\sum_{i=1}^{N}p_{i}+\sum_{i=1}^{N}t_{i}+\mathrm{smooth}}\right) \end{array}$$

where $p_{i}$ denotes the predicted probability, $t_{i}$ denotes the target label (usually 0 or 1), and *N* is the total number of pixels.

The Dice similarity coefficient (DSC) is used to quantify the similarity between the predicted segmentation and the ground truth, and is defined as

(46) $$\mathrm{DSC}=\frac{2TP}{2TP+FP+FN}.$$

Here, TP, FP, TN, and FN denote true positive, false positive, true negative, and false negative, respectively.

## 3. Experimental Results and Analysis

### 3.1. Comparative Experiments

To systematically evaluate the overall performance of the proposed FLDO-LKNet model, it was compared with several classical and recently developed segmentation approaches, including UNet [40], UNet++ [16], UKan [27], DeepLabv3 [18], VM-Unet [25], MC-Unet [41], AISOA-SSformer [20], and CGWO-LWNet [3]. These methods were selected so that the comparison includes, at three levels of proximity to our task, methods applied to comparable cell- or plant-tissue segmentation, together with broad architectural coverage. First, at the same-task level, CGWO-LWNet is a high-precision method developed specifically for rubber tree stone-cell segmentation, and it serves as the same-domain state-of-the-art baseline that our method is required to surpass. Second, at the adjacent-task level, MC-UNet was developed for cell-level segmentation in microscopy images and is thus the closest cell-segmentation baseline, while AISOA-SSformer was developed for plant leaf-disease segmentation and is thus the closest plant-tissue baseline; both target fine-structure delineation under scale, contrast, and illumination variation analogous to that of stone cells. Third, UNet, UNet++, DeepLabv3, VM-UNet (with the UltraLight VM-UNet backbone), and U-KAN provide breadth across the major segmentation families (classic CNN encoder–decoders, a state-space/Mamba model, and a KAN-based model). All methods were re-implemented and re-trained from scratch on our stone-cell dataset under identical data splits, augmentation, and evaluation protocols, so the comparison reflects their behaviour on our task rather than their original domains. For a fair and reliable comparison, all experiments were performed on the same datasets with the same experimental settings and evaluation metrics:

**Quantitative Comparison:**

As seen in Table 4, for the CNN based methods, UNet and UNet++ achieve a mIoU of 63.03% and 62.69% with just a slight performance difference. It indicates that the benefits of dense skip connections and structural refinement in UNet family are limited for the stone cell segmentation task. This limitation is mainly due to the large differences in morphology and scale of stone cells, which tend to form small clusters and are easily confused with background textures, resulting in the existence of both false positive and false negative and limiting the further improvement of mIoU and DSC. UKan has a relatively high accuracy of 96.35%, but its mIoU is still 63.58%, which means that higher per-pixel accuracy does not necessarily ensure accurate spatial overlap between the predicted stone cell regions and the ground truth annotations. When the contrast between stone cells and background is limited due to staining variability or the presence of cellular debris, the model is more prone to false negatives, which further hinders improvement in mIoU.

For the Transformer or hybrid architecture-based methods, DeepLabv3 and AISOA-SSformer obtain improved mIoU of about 67% at 67.85% and 67.54% respectively with 78.58% and 79.58% Sensitivity values, exhibiting better recall of stone cell regions with more semantic information added. However, their mIoU values of 67.85% and 67.54%, together with DSC values of 80.02% and 80.62%, remain below those of the proposed method, indicating that under conditions of complex cell boundaries and fragment interference, enhancing global semantic representation alone, without explicitly suppressing fragment responses, is insufficient to achieve accurate segmentation.

In contrast, the proposed FLDO-LKNet attains the best overall performance, achieving an mIoU of 75.18% and a DSC of 82.36%, which surpass the current state-of-the-art CGWO-LWNet with mIoU and DSC values of 69.10% and 81.70%, corresponding to improvements of 6.08 and 0.66 percentage points, respectively. At the same time, Sensitivity increases from 80.04% to 82.21%, while Specificity remains high at 98.54%, close to the maximum value of 98.80%, indicating that the performance gains are not achieved through excessive expansion of foreground regions, but rather through a further reduction in false negatives and improved region overlap quality while maintaining a low false positive rate, making the proposed method more suitable for stone cell segmentation under irregular morphology, weak contrast, and strong fragment interference. Notably, because foreground pixels occupy only about 5.3% of the image on average, Accuracy and Specificity are close to saturation for all compared methods (94–99%) and thus differ little across models; the substantial and consistent gains in mIoU, DSC, and Sensitivity are therefore the metrics that meaningfully distinguish the methods under this strong class imbalance.

Beyond accuracy, Table 4 also reports the parameter count and computational cost of each method, including the UltraLight VM-UNet baseline. FLDO-LKNet requires only 0.26 M parameters and 1.28 GFLOPs: apart from its own UltraLight VM-UNet backbone (0.049 M/0.24 G), it is the most lightweight of all compared methods (e.g., 39.63 M/172.80 G for DeepLabv3, 27.43 M/16.44 G for VM-UNet, and 1.02 M/5.92 G for CGWO-LWNet), while still achieving the highest segmentation accuracy. Relative to the backbone, the LDFE and KCFA modules add only a modest overhead (0.049→0.26 M parameters, 0.24→1.28 GFLOPs), and, since FLDO operates purely during training and introduces no additional parameters or computation at inference, they are the only modules that add any inference-time cost; together the three modules raise the mIoU from 62.90% to 75.18% (a gain of 12.28 percentage points). This favourable accuracy–efficiency trade-off indicates that the model is readily deployable for large-scale phenotyping on standard, resource-limited hardware.

**Figure 5:**
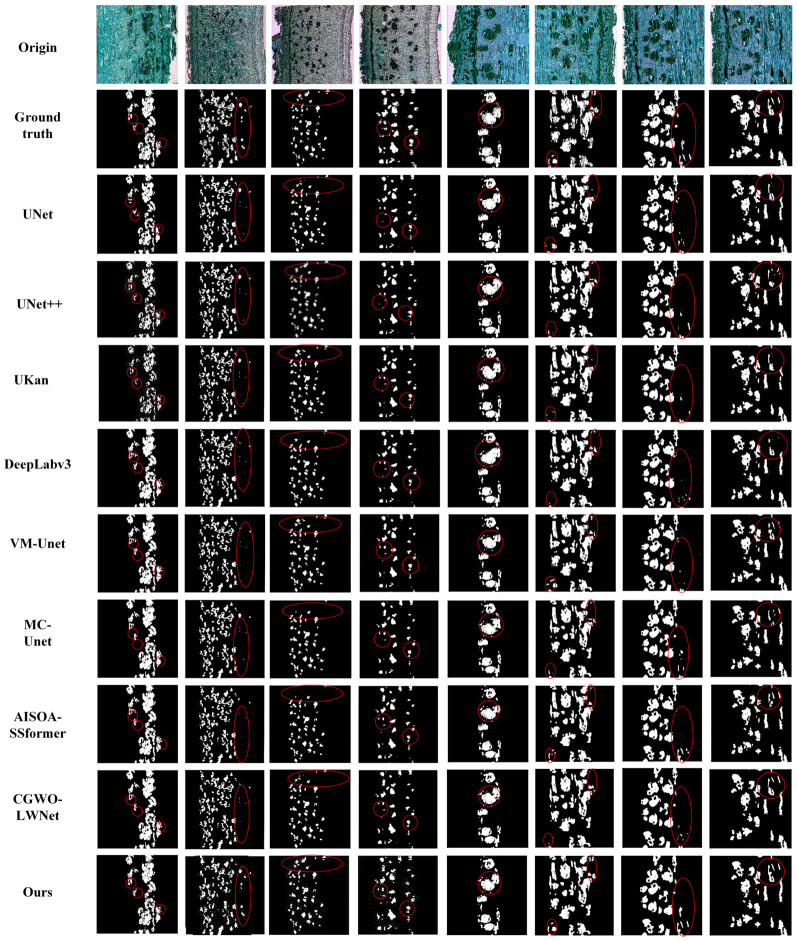
Segmentation performance comparison across different methods.

**Qualitative Comparison:** As shown in Figure 5, stone cell regions with small and scattered fragments as well as interference from cell debris are marked using ellipses, while segmentation errors, missed regions, and redundant predictions are highlighted with small circles. Within the ellipse-marked regions, pure CNN-based methods such as UNet and UNet++ show limited capability in stabilizing fine target details, often resulting in missed detections and blurred contours. Even though UKan and DeepLabv3 can recall some of the stone cell targets, they still have issues with target merging or local omission in low contrast situations, which results in inaccurate representation of complex annotated contours and incomplete segmentation regions. Boundary areas represented by small circles often suffer from over-smoothing, boundary expansion or internal voids, which makes it difficult to obtain accurate alignment with the ground truth, predictions made by other methods.

On the contrary, the proposed method is capable of suppressing the interference of fragments along the boundary of the cell and preserve small stone cell structures under different staining conditions, and reduce the effect of fusion and fragmentation by emphasising contour and structural information and obtain a higher regional overlap quality. As a result, the proposed approach improves the reliability and efficiency of measuring key phenotypic indicators such as stone cell area proportion, which could provide a stable data basis for subsequent phenotyping-related and genetic studies of stone cell traits, and may potentially assist trait evaluation and selection in rubber tree breeding; such downstream analyses, however, are beyond the scope of the present segmentation study and are not evaluated here.

### 3.2. Model Evaluation and Ablation Study

After the comparative experiments above have confirmed the overall superiority of FLDO-LKNet, this section further evaluates the contribution of each proposed component. We first examine the effectiveness of each individual module (LDFE, KCFA, and FLDO) against representative alternatives, and then conduct a comprehensive ablation study to verify the necessity and complementarity of the three modules.

#### 3.2.1. Effectiveness of LDFE

To evaluate the effectiveness of the proposed LDFE module, we replaced it with several representative feature enhancement modules for comparative analysis, including FEM [32], AFE [33], CACEM [34], a convolutional feature transformation module simplified from RepVGG [42], and a local self-attention feature module adapted from Swin Transformer Tiny [43]. Group ① of Table 5 summarises the quantitative results. The model with LDFE achieves an average IoU of 0.752, which is better than all the five comparison modules, indicating that the LDFE provides more effective feature refinement for rubber tree stone-cell segmentation under the conditions of staining variation and fragmented interference. Because FLDO and all other components are held identical across the six variants in this comparison, it is a controlled comparison that isolates the effect of the feature-enhancement module choice rather than that of FLDO; the marginal contribution of LDFE alone is instead quantified in the ablation study (Table 6), where adding LDFE to the baseline with FLDO and KCFA disabled improves mIoU from 62.90% to 64.50%. It should be clarified that LDFE does not assume that stain intensity, illumination, and tissue-preparation effects are physically separable, nor does it attempt to correct each of them independently. Instead, it uses the first- and second-order per-channel statistics as a compact proxy for the combined appearance shift these factors induce on the feature distribution, and learns a channel-wise linear recalibration to compensate for their aggregate effect. Accordingly, the effectiveness of LDFE is validated end-to-end through the above module-comparison ablation rather than by isolating the individual physical factors.

From the structural point of view, FEM has relatively strong local enhancement but it tends to enhance the non-target responses when there is fragmented interference. AFE can perform feature reweighting, but is more sensitive to background drift during fusion for large scale variation and weak boundary contrast. CACEM mainly focuses on channel-wise feature modelling, thus offering limited improvement for accurate boundary localisation.

The RepVGG-based convolutional transformation relies on fixed receptive fields, which reduces its adaptability to multi-scale morphological variation. Although the Swin-style local self-attention module introduces contextual information, it may lead to over-smoothing or inaccurate attention in regions with similar textures, thereby weakening contour consistency. In contrast, the proposed LDFE module effectively suppresses interfering responses while enhancing discriminative features, resulting in more stable and accurate segmentation performance.

#### 3.2.2. Effectiveness of KCFA

To systematically assess the effectiveness of the proposed KCFA module within the network, it was compared with several representative attention mechanisms, including ECA, SCB, SE, and Wave-SC, and the corresponding results are reported in Group ② of Table 5. The experimental results indicate that the model equipped with KCFA achieves the best overall performance in terms of mIoU, DSC, and Sensitivity, while also obtaining the lowest BCE-DSC loss. In contrast, channel attention methods such as SE mainly rely on global feature statistics and therefore tend to amplify background channel responses under strong fragment interference, whereas SC-type spatial attention is highly sensitive to local saliency and may incorrectly focus on fragment noise as target regions, leading to the introduction of false boundaries.

Although the compared attention mechanisms enhance features from channel, spatial, or multi-scale perspectives, they lack effective constraints on the intensity of cross-layer detail injection, which can result in over-enhancement and contour discontinuities in the presence of blurred boundaries or strong noise. By comparison, KCFA improves scale adaptability through multi-scale information interaction and introduces Kalman-style prior updates during cross-layer feature fusion, allowing the module to adaptively adjust detail weights based on decoder structural information, thereby suppressing fragment-induced false responses, reducing false boundaries, preserving contour continuity, and ultimately achieving more stable segmentation performance.

#### 3.2.3. Effectiveness of FLDO

To assess the effectiveness of the proposed FLDO optimizer, we compared it with several representative optimization methods, including Adam, Adamax, AdamW, RMSprop, SGD, and CGWO. For a fair comparison, the learning rate of each optimizer was individually tuned by searching within the range $\left[10^{-5},10^{-2}\right]$, and every configuration was run five times with independent random seeds (0–4), with the results reported as mean ± standard deviation in Group ③ of Table 5. FLDO achieves the best performance among all compared optimisers in terms of BceDSCLoss, mIoU, and DSC. Moreover, FLDO attains the smallest standard deviation on all four metrics (e.g., ±0.005 in mIoU, compared with ±0.007 to ±0.022 for the other optimisers), providing quantitative evidence that its performance-feedback-based learning-rate regulation yields more stable training rather than merely a higher single-run score. The results also demonstrate that its dynamic learning rate regulation strategy based on performance feedback can effectively stabilise gradient updates and accelerate convergence, especially when the stone-cell structures are fragmented and their boundaries are blurred.

FLDO improves the Sensitivity only marginally over CGWO. However, FLDO achieves significantly better mIoU and DSC, suggesting better region overlap and contour localisation. Most probably this advantage can be attributed to the dual-feedback mechanism of FLDO which simultaneously exploits mIoU and DSC to regularise the optimisation. In this way, the boundary expansion can suppress false positives and false negatives simultaneously and avoid excessive segmentation, and thus more reliable segmentation results can be obtained under the conditions of fragment interference and weak-boundary.

In contrast, traditional optimisers are more susceptible to learning-rate selection and gradient noise in this task. Even adaptive methods with better flexibility of updates, such as AdamW and Adamax, can have unstable or insufficient updates in boundary-sensitive regions. Instead, FLDO achieves a more robust segmentation performance under complex boundary conditions by feedback-guided learning-rate adjustment and more stable gradient update behaviour.

**Figure 6:**
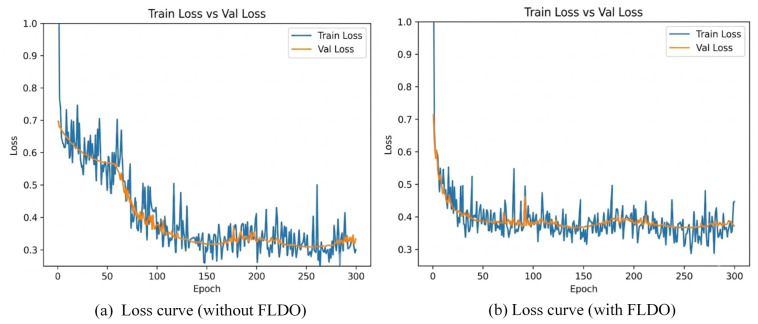
Training and validation loss curves with and without FLDO.

To evaluate the robustness of the proposed FLDO optimiser in the case of serious cell fragment interference, we selected stone cell images with severe boundary fragmentation from the original dataset to construct a fragment interference evaluation subset. As shown in Figure 6, the introduction of FLDO leads to a faster decrease in both training and validation losses, accompanied by smoother loss curves. The magnitude of fluctuations and oscillations during the training process is notably reduced, and convergence reaches a stable plateau at an earlier stage, indicating that FLDO improves training stability and accelerates convergence in scenarios affected by fragment interference.

Finally, since the primary motivation for FLDO is to reduce the sensitivity to the initial learning rate caused by fragment interference, we conduct a dedicated sensitivity analysis. We train FLDO-LKNet under three fixed initial learning rates spanning one order of magnitude ($3\times 10^{-4}$, $1\times 10^{-3}$, and $3\times 10^{-3}$), each with and without FLDO, and report the resulting test-set mIoU in Table 7. Without FLDO, the mIoU varies by 5.84 percentage points across this range, whereas with FLDO the spread is only 1.13 percentage points; FLDO thus clearly flattens the performance curve and desensitizes training to the initial learning rate. Notably, the “without FLDO” setting corresponds to AdamW, whose learning rate was already individually tuned over $\left[10^{-5},10^{-2}\right]$, yet every FLDO run—including those at deliberately sub-optimal learning rates—still exceeds its best-tuned result (72.02%). Moreover, cosine annealing applies a fixed, pre-scheduled decay that is independent of the optimization state and therefore cannot detect or recover from a poorly chosen initial learning rate, whereas FLDO regulates the learning rate online from validation-performance feedback; the two mechanisms are orthogonal and could in principle be combined. These results establish that the advantage of FLDO is not reproducible by simply tuning AdamW or adding a cosine schedule.

#### 3.2.4. Ablation Study

**Figure 7:**
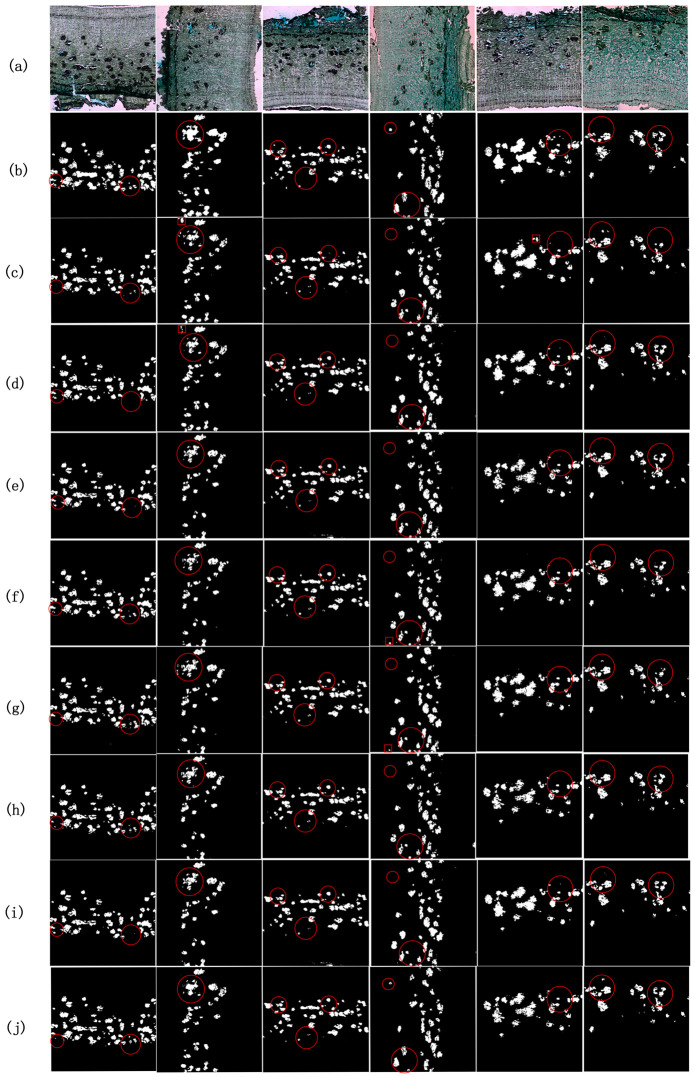
Ablation study visualization: (**a**) original image; (**b**) ground truth; (**c**) UltraLight VM-UNet; (**d**) LDFE; (**e**) KCFA; (**f**) FLDO; (**g**) LDFE + FLDO; (**h**) KCFA + FLDO; (**i**) LDFE + KCFA; (**j**) FLDO-LKNet.

To validate the effectiveness of the proposed FLDO-LKNet, systematic ablation experiments were conducted using UltraLight VM-UNet as the baseline model. Eight different configurations were designed by progressively incorporating the LDFE, KCFA, and FLDO modules, and all experiments were performed with a fixed training epoch number of 300 and a batch size of 8 under identical hardware and software environments to ensure fair and reliable comparisons. The qualitative visual results and quantitative performance metrics presented in Figure 7 and Table 6 collectively illustrate the influence of each module on the overall model performance. The experimental results show that the introduction of LDFE, KCFA, and FLDO consistently leads to performance improvements, confirming the effectiveness of each individual component. After fully integrating the LDFE, KCFA, and FLDO modules into the backbone network, the model achieves the best performance on the test set, with the mIoU reaching 75.2% and the DSC reaching 82.36%, which outperforms all other ablation configurations and demonstrates the overall effectiveness of the proposed module combination.

## 4. Discussion

In this study, we compared the proposed FLDO-LKNet with eight mainstream networks, including UNet, UNet++, UKan, DeepLabv3, VM-Unet, MC-Unet, AISOA-SSformer, and CGWO-LWNet. Experimental results demonstrate that our model consistently outperforms all competitors across key metrics such as mIoU, Accuracy, and DSC. Notably, compared with the state-of-the-art CGWO-LWNet, our model improves mIoU by 6.08 percentage points, from 69.10% to 75.18%, and increases Sensitivity by 2.17 percentage points, from 80.04% to 82.21%, validating the superior performance of our approach in rubber tree stone cell segmentation.

The overall success of FLDO-LKNet stems from its specialized design that harmonizes biological characteristics with deep learning architectures. By synergistically integrating **LDFE** for feature recalibration, **KCFA** for cross-layer detail fusion, and **FLDO** for training optimization, the model establishes a robust framework capable of extracting fine-grained textures and precise boundaries under complex conditions. This holistic integration transforms rubber tree bark analysis from a labor-intensive manual task into a high-efficiency automated workflow, offering a reliable and scalable segmentation tool with the potential to support downstream applications such as variety improvement and cultivation management, which are beyond the scope of the present study and are not evaluated here.

We further assess the feasibility of deploying FLDO-LKNet in real-world breeding scenarios in terms of memory usage and inference speed, which are directly characterized by the two hardware-independent efficiency metrics reported for all methods in Table 4: the parameter count and the computational cost (FLOPs). In terms of memory, the model contains only 0.26 M parameters, corresponding to a weight-storage requirement of roughly 1 MB in single precision, which is negligible even for embedded or edge devices. In terms of speed, FLOPs is the primary hardware-independent determinant of inference latency, and FLDO-LKNet requires only 1.28 GFLOPs—apart from its own UltraLight VM-UNet backbone, the lowest among all compared methods (e.g., 172.80 G for DeepLabv3 and 16.44 G for VM-UNet)—so it is among the fastest models in the comparison and its inference cost is orders of magnitude lower than manual annotation. We report parameter count and FLOPs rather than wall-clock latency and peak memory because the former are reproducible and independent of the specific hardware, batch size, framework version, and I/O conditions, allowing a fair and transferable assessment across deployment environments. Because FLDO operates only during training, these inference-time costs are not affected by the optimizer. Overall, the compact size and low computational cost indicate that FLDO-LKNet is readily deployable on standard, resource-limited hardware for large-scale, high-throughput stone-cell phenotyping; on-device/edge deployment and further model compression (e.g., quantization and pruning) are left as directions for future work.

However, despite its achievements, the model faces a few limitations due to its task-specific design. In particular, the modules’ dependency on context aggregation and feedback mechanisms might be affected by performance degradation under extreme morphological noise or non-linear illumination distortions. Moreover, the robustness of the optimiser and its dependence on hyperparameters need to be further validated on larger and more diverse datasets. Furthermore, while the current dataset is high-quality, its limited sample size across different growth stages may constrain the model’s generalization ability in highly varied real-world scenarios. In addition, the training, validation, and test partitions in this study were performed at the image level. Although near-duplicate adjacent serial sections of the same sample were removed during dataset curation (Section 2.2), we cannot fully rule out that images originating from the same plant, clone, slide, or microscope appear in different subsets, which may introduce a degree of optimistic bias. A strict group-wise partition that isolates the subsets at the plant, clone, and slide level is therefore an important direction for future work to further validate the generalization of the model. Moreover, the photometric data augmentation in this study was limited to brightness variation; systematically incorporating contrast, saturation, and colour-jitter augmentation, together with dedicated stain-normalization strategies, would provide a broader experimental basis for the model’s robustness to staining and contrast shifts. Finally, LDFE compensates for the aggregate appearance shift captured by per-channel statistics, and the current experiments do not isolate stain intensity, illumination, and tissue preparation as independent factors; a controlled factor-isolation study, based on data in which these variables are varied one at a time, would allow a more fine-grained assessment of what LDFE actually corrects and is left for future work. It should also be noted that, although the independent test set already spans a certain degree of acquisition diversity—covering three microscope–camera combinations (Olympus BX53 with VP700c, Olympus CX43 with LV2000, and Leica DMBL with Leica DFC550), two clonal populations and one hybrid progeny population, and multiple planting and sampling years—all images were ultimately collected from a single geographic source (CATAS, Hainan). Consequently, the current evaluation does not include a truly independent cross-site test or samples prepared in a separate laboratory batch, which limits the assessment of cross-domain generalization. Supplementing the evaluation with cross-site and different batch-preparation data to further verify robustness is an important direction for future work.

Future work will focus on two main directions to address these challenges. First, we aim to expand the scale and diversity of the dataset while exploring semi-supervised learning to reduce reliance on fine annotations. Second, we will investigate the “cross-task generalization” of FLDO-LKNet by integrating its core components into mainstream frameworks for broader agricultural tasks, such as pest detection and fruit counting. In particular, extending the method from controlled histological images to unstructured field environments (e.g., orchard scenes with variable illumination, occlusion, and complex backgrounds) will require dedicated robustness strategies such as those systematically reviewed for fruit detection by Tang et al. [44], which provide a valuable reference for adapting our modules to in-field agricultural vision. In addition, we plan to instrument per-step logging of the FLDO agent’s internal Q-table so as to characterize its learning dynamics in detail—for example, the epoch-wise |ΔQ| convergence and the number of validation cycles required for the greedy policy to stabilize—which was not recorded in the present experiments. Through these efforts, we seek to define the applicability boundaries of our proposed strategies and provide generalizable references for model design in complex agricultural visual analysis.

## 5. Conclusions

In this study, to address the challenges of insufficient segmentation accuracy caused by complex edge morphology, variable scales, staining differences, and cell fragment interference in rubber tree stone cells, we propose an efficient segmentation method, FLDO-LKNet. Compared with the existing state-of-the-art method, FLDO-LKNet improves mIoU and Sensitivity by 6.08 and 2.17 percentage points, respectively, demonstrating superior segmentation performance.

First, to mitigate the interference of staining variation, we introduce LDFE. This method utilizes channel-level adaptive linear recalibration to compensate for illumination and staining biases, thereby reducing the model’s reliance on unstable appearance cues and promoting greater focus on texture and other detailed features.

Second, to address the insufficient boundary delineation caused by the complex morphology and large scale variations of stone cells, we propose the KCFA mechanism. This mechanism enhances boundary response by integrating multi-scale representations with joint channel-spatial modeling. A Kalman-style cross-layer fusion strategy then employs decoder semantic priors to guide encoder detail observations, refining and completing the complex boundaries of stone cells under semantic constraints.

Finally, to mitigate learning rate sensitivity and training instability caused by noisy samples such as cell fragments, we introduce FLDO. Building upon adaptive gradient estimation, FLDO incorporates a performance-feedback-driven dynamic learning rate regulation mechanism, improving optimization stability and convergence reliability.

Ablation studies further demonstrate that LDFE, KCFA, and FLDO all contribute to the improvement of segmentation performance, with the complete FLDO-LKNet achieving the highest mIoU of 75.2% and DSC of 82.36%. Additionally, the module-comparison ablations validate the respective contributions of LDFE and KCFA to handling staining variations and morphological and scale changes, while a dedicated fragment-interference subset further confirms the advantage of FLDO in stabilizing training dynamics.

In summary, the proposed FLDO-LKNet model demonstrates exceptional segmentation performance on rubber tree stone cell images characterized by significant staining variations and substantial morphological and scale differences. By automating the segmentation process, this model provides a reliable tool that replaces labor-intensive manual analysis with high-efficiency automation. As the present study focuses on the segmentation task itself, it establishes an accurate and scalable quantification of stone-cell phenotypes that can serve as a foundation for downstream applications such as variety improvement, cultivation optimization, and quality evaluation of rubber. We note, however, that these downstream applications are not experimentally validated in this work: no phenotype–trait correlation or breeding analysis is conducted here. Systematically linking the extracted stone-cell phenotypes to agronomic and genetic traits is left as an important direction for future work.

## Figures and Tables

**Table 1:** Algorithmic Framework of FLDO.

Algorithm FLDO Optimizer
**Require:** Parameters θ, base learning rate $\alpha_{0}$, hyper-parameters Ω **Ensure:** Updated parameters θ 1: Initialize m←0, v←0, lr_scale←1 2: Initialize Q(s,a)←0 for all states *s* and actions *a* 3: **for** t←1,2,… **do** 4: Sample mini-batch $B_{t}$ 5: Compute loss $L_{t}\leftarrow L\left(B_{t};\theta \right)$ 6: $g_{t}\leftarrow \nabla_{\theta }\;L_{t}$ 7: $m\leftarrow \beta_{1}m+\left(1-\beta_{1}\right)g_{t}$ 8: $v\leftarrow \beta_{2}v+\left(1-\beta_{2}\right)\;\left(g_{t}-m\right)⊙\left(g_{t}-m\right)$ 9: $\hat{m}\leftarrow m/\left(1-\beta_{1}^{t}\right)$, $\hat{v}\leftarrow v/\left(1-\beta_{2}^{t}\right)$ 10: $\hat{g}\leftarrow \hat{m}/\left(\sqrt{\hat{v}}+\epsilon \right)$ 11: **for** b←1,…,B **do** 12: $u_{b}\leftarrow \phi_{b}\left(\hat{g}\right)$ 13: **end for** 14: $w\leftarrow \mathrm{Softmax}\left(\mathrm{Score}(u_{1},\ldots ,u_{B})\right)$ 15: $\tilde{g}\leftarrow \sum_{b}w_{b}\;u_{b}$ 16: $\theta \leftarrow \left(1-\alpha_{0}\cdot lr\_scale\cdot \lambda \right)\;\theta -\alpha_{0}\cdot lr\_scale\cdot \tilde{g}$ 17: **if** $t\;\mathrm{mod}\;T_{\mathrm{val}}=0$ **then** 18: $\left(I_{t},D_{t}\right)\leftarrow \mathrm{Validate}\left(\theta \right)$ 19: $r_{t}\leftarrow \left(I_{t}-I_{t-T_{\mathrm{val}}}\right)+\left(D_{t}-D_{t-T_{\mathrm{val}}}\right)$ 20: $s_{t}\leftarrow \mathrm{State}\left(m,v,L_{t},L_{t-1},\mu_{t},\sigma_{t}^{2}\right)$ 21: $a_{t}\leftarrow \epsilon \text{-}\mathrm{greedy}\left(Q,s_{t}\right)$ 22: $lr\_scale\leftarrow \rho (a_{t})$ 23: $s_{t+1}\leftarrow \mathrm{State}\left(m,v,L_{t+1},L_{t},\mu_{t+1},\sigma_{t+1}^{2}\right)$ 24: $Q\left(s_{t},a_{t}\right)\leftarrow \left(1-\eta_{Q}\right)Q\left(s_{t},a_{t}\right)+\eta_{Q}\left[r_{t}+\gamma_{Q}\max_{a^{\prime }}Q\left(s_{t+1},a^{\prime }\right)\right]$ 25: **end if** 26: **end for**

**Table 2:** Hyperparameter settings of the reinforcement-learning component in FLDO.

Hyperparameter	Symbol	Value
Q-learning update rate	$\eta_{Q}$	0.1
Discount factor	$\gamma_{Q}$	0.9
Validation interval	$T_{\mathrm{val}}$	94 iterations (1 epoch)
Action space size	|A|	5
LR scaling factors	A	{0.5,0.8,1.0,1.25,1.5}
Exploration policy	ϵ-greedy	0.5→0.05 (τ=100)
Base learning rate	$\eta_{0}$	$1\times 10^{-3}$

**Table 3:** Experimental platform and runtime configuration.

Category	Configuration
Computing platform	AMD EPYC 9754 128-Core Processor, allocated with 22 virtual CPU cores
Graphics accelerator	NVIDIA GeForce RTX 4090 with 24 GB memory
Operating environment	Ubuntu 20.04 with Python 3.8
GPU computing stack	CUDA Toolkit 11.8
Deep learning acceleration library	cuDNN 8.6.0

**Table 4:** Performance comparison of stone cell segmentation methods in rubber tree bark. FLOPs are measured at an input resolution of 512×512. The best result in each column is highlighted in bold.

Method	mIoU (%)	Accuracy (%)	Sensitivity (%)	Specificity (%)	DSC (%)	Params (M)	FLOPs (G)
UNet	63.03	94.11	76.32	93.81	77.66	7.77	55.04
UNet++	62.69	94.45	73.28	94.15	77.50	9.16	139.60
UKan	63.58	96.35	76.59	97.06	77.81	6.36	6.00
DeepLabv3	67.85	95.95	78.58	95.28	80.02	39.63	172.80
VM-UNet	66.64	97.62	76.41	97.41	79.98	27.43	16.44
MC-UNet	65.81	96.59	78.92	98.76	79.38	4.58	32.96
AISOA-SSformer	67.54	97.71	79.58	98.80	80.62	2.31	12.20
CGWO-LWNet	69.10	97.81	80.04	98.61	81.70	1.02	5.92
UltraLight VM-UNet	62.90	95.22	77.25	96.55	77.23	0.049	0.24
**Ours**	**75.18**	**98.50**	**82.21**	**98.54**	**82.36**	**0.26**	**1.28**

**Table 5:** Results of FLDO-LKNet module experiment. Entries in Group ③ (optimizer comparison) and the final row (Ours) are reported as mean ± standard deviation over five independent runs with random seeds 0–4; the remaining entries correspond to a single representative run. The best result in each column is highlighted in bold.

Group	Method	Loss	mIoU	DSC	Sensitivity
①	FEM	0.326	0.697	0.798	0.772
	AFE	0.371	0.573	0.681	0.623
	CACEM	0.332	0.624	0.772	0.791
	Swin Transformer Tiny	0.329	0.653	0.781	0.754
	RepVGG	0.331	0.693	0.803	0.787
②	ECA	0.332	0.657	0.793	0.772
	SCB	0.371	0.651	0.788	0.804
	SE	0.326	0.676	0.780	0.801
	Wave-SC	0.312	0.673	0.814	0.805
③	Adam	0.379±0.012	0.639±0.011	0.780±0.009	0.805±0.014
	Adamax	0.394±0.013	0.629±0.012	0.772±0.010	0.801±0.015
	AdamW	0.369±0.010	0.651±0.009	0.789±0.008	0.810±0.012
	RMSprop	0.378±0.013	0.643±0.012	0.783±0.010	0.795±0.014
	SGD	0.525±0.030	0.516±0.022	0.681±0.018	0.642±0.025
	CGWO	0.315±0.008	0.654±0.007	0.812±0.006	0.821±0.010
	**Ours**	0.301±0.006	0.752±0.005	0.824±0.004	0.822±0.008

**Table 6:** Ablation study results. The best result in each column is highlighted in bold.

LDFE	KCFA	FLDO	Loss	mIoU	DSC	Sensitivity
baseline			0.395	0.629	0.772	0.772
✓			0.315	0.645	0.811	0.799
		✓	0.313	0.667	0.818	0.802
✓		✓	0.312	0.651	0.814	0.808
	✓		0.311	0.697	0.819	0.806
	✓	✓	0.309	0.729	0.821	0.805
✓	✓		0.305	0.716	0.802	0.810
✓	✓	✓	**0.301**	**0.752**	**0.824**	**0.822**

**Table 7:** Learning-rate sensitivity of FLDO-LKNet, reported as test-set mIoU (%). The spread (max–min) across the learning-rate range drops from 5.84 to 1.13 points when FLDO is enabled. The best result is highlighted in bold.

Initial Learning Rate	w/o FLDO	w/FLDO
$3\times 10^{-4}$	68.35	74.21
$1\times 10^{-3}$	72.02	**75.18**
$3\times 10^{-3}$	66.18	74.05
Spread (max–min)	5.84	**1.13**

## Data Availability

The data supporting the findings of this study are available in an anonymized GitHub repository at: https://anonymous.4open.science/r/Rubber-tree-stone-cells-C307 (accessed on 9 July 2026).
